# The Effect of Occupational Lead Exposure on Blood Levels of Zinc, Iron, Copper, Selenium and Related Proteins

**DOI:** 10.1007/s12011-012-9490-x

**Published:** 2012-08-26

**Authors:** Aleksandra Kasperczyk, Adam Prokopowicz, Michał Dobrakowski, Natalia Pawlas, Sławomir Kasperczyk

**Affiliations:** 1 Department of Biochemistry, Medical University of Silesia in Katowice, Jordana 19, 41-808 Zabrze, Poland; 2 Department of Chemical Hazards and Genetic Toxicology, Institute of Occupational Medicine and Environmental Health, Kościelna13, 41-200 Sosnowiec, Poland

**Keywords:** Lead poisoning, Trace elements, Acute-phase proteins, Haptoglobin, Caeruloplasmin, Transferrin

## Abstract

The study objective was to evaluate the effect of occupational lead exposure on blood concentrations of zinc, iron, copper, selenium and proteins related to them, such as transferrin, caeruloplasmin and haptoglobin. The examined group consisted of 192 healthy male employees of zinc–lead works. By the degree of lead exposure, the exposed group was subdivided into three subgroups. The control group was composed of 73 healthy male administrative workers. The markers of lead exposure (blood levels of lead and zinc protoporphyrin) were significantly elevated in the exposed group compared with the control group. Additionally, concentrations of copper and caeruloplasmin were raised. The significant increase in haptoglobin level was observed only in the low exposure group. Selenium levels were significantly decreased, whereas iron, zinc and transferrin levels were unchanged in the exposed group compared with the control group. There were positive correlations between the lead toxicity parameters and the copper and caeruloplasmin levels. In conclusion, the effect of occupational exposure to lead on the metabolism of trace metals appears to be limited. However, significant associations between lead exposure and levels of copper and selenium were shown. Changed levels of positive acute-phase proteins, such as caeruloplasmin and haptoglobin, were also observed.

## Introduction

Although lead induces physiological, biochemical and behavioural disturbances in humans, exposure to this xenobiotic is unavoidable because of its accumulation in the environment and use in industrial applications [1, 2]. Being present in contaminated water, air, food and dust [3], lead is mostly absorbed by the lungs and gastrointestinal tract, whereas percutaneous absorption of inorganic lead is minimal. In adults, 40–50 % of inhaled and approximately 10 % of ingested lead are transferred to the bloodstream and then distributed by plasma throughout the soft tissues and bones [4].

One of the major targets for lead toxicity is the thiol group of enzymes. Consequently, lead has an inhibitory effect on delta-aminolevulinic acid dehydratase. Because lead also inhibits ferrochelatase, it impairs the chain reaction that leads to the formation of haem; this impairment results in anaemia and the accumulation of delta-aminolevulinic acid (ALA) and zinc protoporphyrin (ZPP) in erythrocytes [5, 6]. Concentrations of ALA and ZPP are used as biomarkers of human lead exposure [4].

Lead is a redox inactive metal [5]. However, lead has pro-oxidative activity and can generate reactive oxygen species (ROS) and reduce cell antioxidant defences, such as antioxidant enzymes and glutathione [2]. Moreover, ALA that accumulates in saturnism has pro-oxidant properties [7].

Furthermore, lead interacts with some essential metals [2]. One of them is selenium (Se), which plays an important role as an antioxidant [8]. Se is a cofactor of glutathione peroxidase, decreases the amount of lipid peroxidation and protects DNA, RNA and proteins from oxidative damage. Additionally, Se forms inactive selenium–lead complexes [9] and, consequently, reduces the availability of free lead ions in the body [10].

A lead–zinc interaction has been observed [11]. Zinc (Zn) is essential for cellular membrane integrity and metabolism [2] as a central part of over 300 enzymes and proteins [12]. Similar to Se, Zn has been shown to possess antioxidant properties caused by its requirement for superoxide dismutase (SOD) activity [13]. Therefore, Zn not only reduces lead-induced oxidative stress but also competes with lead for similar binding sites [11]. Competitive binding to metallothionein-like transport protein in the rat duodenum suggests the ability of Zn to reduce lead absorption [11, 14].

Additionally, copper (Cu) has been reported to bind to metallothionein-like transport proteins [14]. Cu is contained in caeruloplasmin, an α_2_-globulin having enzymatic properties, and is responsible for the oxidation of ferrous to ferric iron and catalyses the transport of iron to transferrin, which transfers bound ions to cells. Because lead binds to both caeruloplasmin and transferrin, iron (Fe) metabolism in exposed individuals could be impaired [15, 16]. Another antagonism between these metals may occur in the intestine because low dietary intake of Fe should increase the absorption of lead [17]. Moreover, limited Fe in the mitochondria may enhance lead-induced haem synthesis inhibition [11].

In short, the presence of metals, such as Se, Zn, Cu and Fe, modifies lead toxicity, but their interactions are unclear.

Therefore, the present study was undertaken to determine the effect of occupational exposure to lead on blood levels of the above-mentioned trace metals and proteins that are related to them.

## Materials and Methods

### Study Population

The experimental protocol has been approved by the Bioethics Committee of the Medical University of Silesia in Katowice no. NN-6501-36/I/06. The examined group included 192 male employees of zinc and lead works localised in the southern region of Poland with an age range of 22–58 years. The study subjects had been exposed to lead for 4 to 37 years. Workers suffering from chronic diseases were excluded.

To determine the amount of lead exposure (exposure to zinc was insignificant), the concentrations of lead and zinc protoporphyrin in the blood samples were determined, on average, every 3 months during the 2 years of observation. From the collected data, the mean blood concentrations of lead (PbB_mean_) and zinc protoporphyrin (ZPP_mean_) were calculated. In view of the obtained values, the examined population was divided into three subgroups: low exposure to lead (LE), medium exposure to lead (ME) and high exposure to lead (HE). The LE group consisted of 56 workers with PbB_mean_ less than 40 μg/dl. The ME group included 67 workers with a PbB_mean_ from 40 to 50 μg/dl and a ZPP_mean_ from 5 to 7.5 μg/g Hgb, whereas 69 workers with a PbB_mean_ greater than 45 μg/dl and a ZPP_mean_ greater than 7.5 μg/g Hgb were classified as the HE group.

In the last collected blood samples, blood lead level (PbB), blood zinc protoporphyrin level (ZPP) and concentrations of iron, selenium, copper, zinc, caeruloplasmin, haptoglobin and transferrin were measured concomitantly.

The control group consisted of 73 healthy male administrative workers who were exposed to lead only environmentally and had no history of occupational exposure to lead. The age range of the control group was 21 to 60 years. No one from this group had PbB or ZPP levels greater than the normal levels, which were 10 μg/dl and 2.5 μg/g Hgb, respectively.

### Sampling and Laboratory Procedures

By venipuncture, 10 ml of blood was collected into plain tubes to obtain serum, whereas 15 ml was placed in tubes containing an ethylenediaminetetraacetic disodium acid solution as an anticoagulant to obtain plasma and erythrocytes.

Whole blood was used for the analysis of PbB and ZPP. The determination of PbB was performed by graphite furnace atomic absorption spectrophotometry, using Unicam 929 and 939OZ Atomic Absorption Spectrometers with GF90 and GF90Z Graphite Furnaces. The data were reported in micrograms per deciliter. ZPP was measured using an Aviv Biomedical Hematofluorometer, Model 206. The results were expressed as micrograms per gram of haemoglobin.

After centrifugation of the remaining blood, plasma was separated for zinc, copper and selenium analysis. The sedimented red blood cells were washed three times with 0.9 % NaCl and then lysed with bidistilled water. In 10 % haemolysate, the concentration of haemoglobin was determined using the cyanmethaemoglobin method.

The concentrations of Zn, Cu and Se in plasma were determined by atomic absorption spectrophotometer using an acetylene-air flame. The results were reported in micrograms per deciliter. Serum Fe analysis was performed on A25 Clinical Analyzer (BioSystems, Spain) according to the manufacturer’s instructions. The obtained values were expressed in micromoles per liter.

The concentration of caeruloplasmin in serum was determined by Richterich [18] and expressed in micrograms per deciliter. Serum transferrin and haptoglobin levels were measured by immunoturbidimetric assays. Specific rabbit monoclonal antibodies (Dako-Cytomation, Denmark) were used according to the manufacturer’s instructions. The measurements were performed on Biochemical Analyzer EM 280 (Emapol, Poland).

### Statistical Analysis

The statistical analysis was performed using Statistica 9.1 PL software. The statistical methods included the mean and standard deviation. Shapiro–Wilk’s test was used to verify normality, and Levene’s test was used to verify homogeneity of variances. Either an analysis of variance or Kruskal–Wallis ANOVA test was used for multiple comparisons of data. Additional statistical comparisons were made using either a *t* test, *t* test with separate variance estimates or a Mann–Whitney *U* test. A Spearman non-parametric correlation was calculated. A value of *p* < 0.05 was considered to be significant.

## Results

There were no significant differences in age, body mass index and smoking habits between the examined and control groups (Table 1). Nevertheless, when comparing the control group with the subgroups, the mean age was significantly lower in the low exposure group (LE) by 10.5 %. The mean PbB and ZPP levels were significantly higher in the LE group by 492 and 242 %, respectively, in the medium exposure group (ME) by 650 and 346 %, respectively, and in the high exposure group (HE) by 680 and 420 %, respectively.

**Table 1:** Epidemiologic parameters: the blood lead level (PbB), zinc protoporphyrin concentration in blood (ZPP) and concentrations of iron (Fe), zinc (Zn), copper (Cu) and selenium (Se) in plasma as well as caeruloplasmin (CER), transferrin (TRF) and haptoglobin (HPG) serum levels in the study population (LE—the low exposure group, ME—the medium exposure group, HE—the high exposure group)

	Control group		LE group			ME group			HE group			ANOVA
	Mean	SD	Mean	SD	*p* value	Mean	SD	*p* value	Mean	SD	*p* value	*p* value
Age	41.5	9.23	37.2	8.80	0.007	43.0	10.2	0.382	43.9	7.53	0.102	0.100
Years of work			13.0	9.61		19.3	9.75		19.2	9.45		0.001
BMI (kg/m^2^)	26.2	3.05	26.7	3.46	0.382	26.6	3.92	0.459	27.2	3.69	0.069	0.098
Smokers (%)	47 %		45 %		0.829	49 %		0.753	62 %		0.060	0.270
PbB_mean_ (μg/dl)	6.45	2.49	34.1	5.39	<0.001	43.4	2.35	<0.001	49.7	4.10	<0.001	<0.001
PbB (μg/dl)	6.39	2.47	37.8	10.0	<0.001	47.9	6.75	<0.001	49.8	5.98	<0.001	<0.001
ZPP_mean_ (μg/g Hb)	1.93	0.47	6.03	3.07	<0.001	8.15	4.13	<0.001	10.2	2.41	<0.001	<0.001
ZPP (μg/g Hb)	1.96	0.51	6.68	3.73	<0.001	8.72	5.06	<0.001	10.2	3.10	<0.001	<0.001
Fe (μmol/l)	102	45.8	96.4	35.2	0.418	104	43.0	0.843	106	46.5	0.646	0.804
Se (μg/dl)	80.6	15.0	61.1	13.3	<0.001	63.3	9.83	<0.001	69.7	15.6	0.010	<0.001
Cu (μg/dl)	69.1	8.48	77.3	13.9	0.003	78.5	16.4	0.002	77.4	14.2	0.004	0.028
Zn (μg/dl)	70.4	11.2	75.5	12.9	0.071	74.5	10.4	0.085	72.2	12.2	0.497	0.029
CER (mg/dl)	36.6	12.4	45.0	8.64	<0.001	43.1	11.6	0.003	42.5	10.1	0.003	<0.001
HPG (mg/dl)	117	51.8	153	63.2	0.002	132	58.2	0.212	130	48.4	0.211	0.062
TRF (mg/dl)	253	33.7	269	42.8	0.074	264	47.3	0.235	257	42.3	0.588	0.469

**Fig. 1:**
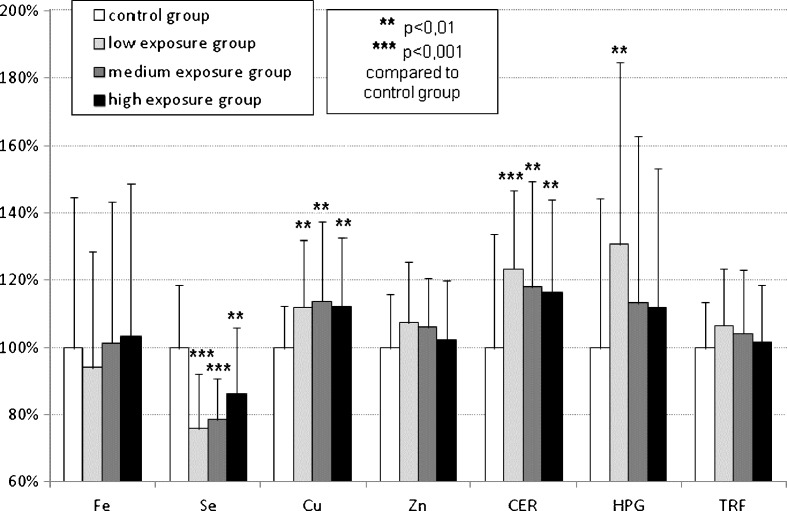
Plasma concentrations of iron (Fe), zinc (Zn), copper (Cu) and selenium (Se) as well as caeruloplasmin (CER), transferrin (TRF) and haptoglobin (HPG) serum levels in lead-exposed groups presented as 100 % of mean ± SD of control

The concentrations of iron, selenium, copper, zinc, caeruloplasmin, haptoglobin and transferrin in the examined subgroups are shown in Fig. 1 as a percentage of the values obtained from the control group.

The copper concentration significantly increased by 11.8 % in the LE group, by 13.6 % in the ME group and by 12.0 % in the HE group compared to the control group. The concentrations of caeruloplasmin increased by 23.2, 17.9 and 16.3 %, respectively, for the LE, ME and HE groups compared to the control group. However, the level of haptoglobin in the LE group was significantly raised by 30.6 %, whereas in the ME and HE groups, only an insignificant tendency to increase was observed. Only the selenium levels were significantly lower by 24.2 % in the LE group, by 21.4 % in the ME group and by 13.6 % in the HE group compared to the control group.

There were no significant changes in iron, zinc and transferrin concentrations in the study population.

The Spearman correlation (Table 2) indicated that there is a positive correlation between the lead toxicity parameters (PbB, ZPP) and copper (*R* = 0.14–0.33) and caeruloplasmin (*R* = 0.14–0.28) levels. There were no correlations with other trace metals and proteins. However, caeruloplasmin correlated positively with copper (*R* = 0.43; *p* < 0.001).

**Table 2:** Correlation between the study parameters (Spearman *R* values, *p* < 0.05, *NS*—non significant)

	Age	Years of work	BMI	PbB mean	PbB last	ZPP mean	ZPP last	Fe	Se	Cu	Zn	CER	HPG
Years of work	0.81												
BMI	0.23	NS											
PbBmean	0.20	0.27	0.16										
PbB	0.23	0.31	NS	0.82									
ZPPmean	0.18	0.24	NS	0.75	0.66								
ZPP	0.14	0.18	NS	0.71	0.71	0.94							
Fe	NS	NS	NS	NS	NS	NS	NS						
Se	NS	NS	NS	NS	NS	NS	NS	NS					
Cu	0.24	0.22	NS	0.14	0.16	0.28	0.33	NS	NS				
Zn	NS	NS	NS	NS	NS	NS	NS	0.25	NS	0.25			
CER	0.23	0.26	NS	NS	0.14	0.28	0.27	NS	NS	0.43	NS		
HPG	0.21	0.22	0.17	NS	NS	NS	NS	NS	NS	0.23	NS	0.27	
TRF	NS	NS	0.18	NS	NS	NS	NS	NS	NS	0.25	NS	NS	0.25

## Discussion

The aim of the study was to evaluate the effect of occupational exposure to lead on blood levels of Zn, Fe, Cu, Se and related proteins, such as transferrin (TRF), caeruloplasmin (CER) and haptoglobin (HPG). The association between lead exposure and levels of Cu and Se was shown. Besides, altered levels of acute-phase proteins, such as CER and HPG, were observed.

The influence of trace metals on lead toxicity has been reported in many animal studies. Klauder and Petering [19] reported that adequate dietary Cu and Fe intake minimises the toxic effect of orally administered lead in rats. Inconsistent results were observed in a study by Cerklewski and Forbes [14], who suggested that high dietary Cu might increase lead toxicity. Conversely, other findings of Cerklewski and Forbes showed that there is a protective effect of dietary Zn and Se on lead toxicity in rats [20, 21]. Similar results have been reported by Batra et al. [22], who observed a significant reduction in lead content in the kidney, liver, spleen, testis, blood and bones because of Zn supplementation. The reports by Bandhu et al. [23] and Prasanthi et al. [24] are in agreement with this study. The protective effect from lead toxicity is attributed to dietary Se [8, 10, 25]; however, when Se was introduced through placental transfer by Sidhu and Nehru [26] or intramuscular injection by Othaman and El Missiry [9], consistent results were obtained.

Animal studies are concordant with those reports that included children populations. Zimmermann et al. [27] improved the Fe status in iron-deficient children exposed environmentally to lead thereby reducing their lead levels by 33 %. Because most environmental lead is absorbed in the intestine, the positive effects of Fe intake in this study might be a result of an iron–lead competitive binding to divalent metal transporter 1 (DMT1). Fe has a higher affinity to DMT1 and could inhibit lead uptake in the intestine [27]. Zn competes with lead analogically, which is in agreement with the results obtained by Ahamed et al. [11], who examined anaemic children environmentally exposed to lead and observed a significant negative correlation between both Fe and Zn blood levels and lead concentration. The Cu concentration in blood was not correlated with this parameter [11]. An association between increased blood lead levels and Fe deficiency was postulated by Muwakkit et al. [28] and Hegazy et al. [29]. Another investigation revealed that blood lead levels were negatively correlated with serum Zn and Se concentrations [30]. Additionally, Diouf et al. [5] demonstrated a negative significant correlation between Se levels and blood lead levels in children who were environmentally exposed to this xenobiotic.

Investigations in adult male workers occupationally exposed to lead are inconsistent. The present study revealed that there is no association between Fe and blood lead levels, which is concordant with previous data [31–34]. However, Kim et al. [35] reported a decrease in the serum Fe level in lead-exposed workers, but a significantly lower dietary Fe intake was observed concurrently. Therefore, to expect that increased lead levels inhibit the uptake of Fe as is postulated by some authors would be unreasonable [28] because workers are exposed to lead primarily through the respiratory tract and competitive binding of lead and Fe or Zn to divalent metal transporters in the intestine should have marginal significance in occupational exposure.

In vitro studies indicate that lead not only impairs Fe binding to TRF [16] but also suppresses its synthesis thus, decreasing mRNA and protein levels [36]. There is no corroboration of this observation in our study because a decrease in TRF level was not observed.

In the present study, there was no significant difference in the Zn plasma levels between the examined and the control groups. Similar results have been reported by Mehdi et al. [34] and Chiba et al. [32], whereas Dioka et al. [37] observed that the Zn blood level decreased by 34 % in artisans who were occupationally exposed to lead. When examining zinc–lead miners, Malekirad et al. [2] observed a positive correlation amongst Zn and lead blood levels and significant elevation of these parameters in the examined workers compared with the control group. Because elevated total antioxidant status and lower DNA damage were also indicated in the examined workers, it is possible to expect that simultaneous exposure to Zn may improve antioxidant defence and, therefore, alleviate lead toxicity.

Se should act analogically. In the present study, the Se plasma level was significantly lower in the workers than in the control group. In addition, lead-exposed smelter workers observed by Gustafson et al. [38] had significantly lower plasma Se levels than the control group. Additionally, there was a significant negative correlation between blood lead level and plasma Se level [38]. The findings of Chiba et al. [32] support our study with the observation that plasma Se levels had a tendency to decrease, whereas the Se concentration in erythrocytes increased significantly with an increasing blood lead level. A possible explanation for this association may be that blood lead, which is predominantly present in erythrocytes [11], forms a complex with Se and reduces the lead level in plasma.

The findings concerning Cu levels are more difficult to interpret than those for Se. Studies by Mehdi et al. [34], Wasowicz et al. [39] and Chiba et al. [32] revealed no association between Cu and lead levels in workers, whereas Cu plasma levels in the present study were significantly higher compared with the control group and correlated positively with lead concentrations. Our earlier studies [40] showed that lead exposure is associated with an elevated activity of superoxide dismutase isoenzyme that contains Cu and Zn (CuZn-SOD) in both serum and erythrocytes. Therefore, an increase in the Cu level, which was observed in the present study, may be caused by increased CuZn-SOD activity. This enzyme is part of the antioxidant defence system and its activity may be elevated because of lead-induced oxidative stress [40, 41]. The increase in plasma Cu levels may also be caused by competitive displacement of the metal from tissues by lead ions. Moreover, lead and Cu compete for binding sites on proteins, such as the ATPase complex [42]. Increased bioavailability of displaced Cu may induce ROS generation via the Fenton reaction and contribute to oxidative stress enhancement.

The concentration of CER, which is an acute-phase protein, increased significantly. Therefore, in part, the increase in plasma Cu level may be secondary to the increase in the CER level. Mongiat et al. [43] obtained similar results in a study of welders and suggested that the increase in CER levels is related to the severity of the oxidative stress and plays an adaptative role. A slight increase in the CER level was observed in rats that were orally administered with lead acetate in a dose of 1,000 mg/l for 4 weeks [44]. Nevertheless, Mehdi et al. [34] and Wasowicz et al. [39] showed that there is no effect of lead exposure on CER levels. In contrast, Leelakunakorn et al. [15] postulated that inhibition of CER oxidase activity was lead mediated. This hypothesis is not in conflict with our study because a decrease in the enzymatic activity of CER does not necessarily mean a reduction in its concentration as a protein.

HPG is the second positive acute-phase protein indicated in our study. The mean HPG serum levels increased significantly in the LE group and insignificantly in the ME and HE groups. The increase in the serum levels of both proteins may be caused by the pro-inflammatory properties of lead [45]. Chang et al. [46] reported that 1 μM lead upregulates the transcription of genes encoding cyclooxygenase-2 (COX-2) and cytosolic phospholipase A_2_ in vascular smooth muscle cells. This causes an elevated PGE_2_ secretion. Consistently, Chou et al. [47] showed that low lead ion concentrations induce inflammation by increasing COX-2 gene expression via the EGFR/NF-κB signal transduction pathway in A431 carcinoma cells. Lead exposure may also be associated with altered levels of interleukin-6, interleukin-10 or tumour necrosis factor-alpha [48, 49]. Additionally, Khan et al. [50] observed increased levels of C-reactive protein (CRP) in lead-exposed workers (PbB = 29.1 μg/dl). There was a strong positive correlation between blood lead and CRP levels in this study (*R* = 0.75).

In the present study, the elevations of CER and HPG levels were higher in the LE group than in the ME or HE groups. The LE group was composed of workers who had been exposed to significantly lower doses of lead for a significantly shorter period. Therefore, our study indirectly supports the hypothesis that reduced lead exposure exerts an immunostimulatory effect, whereas higher exposure may cause immunosuppression [51].

## Conclusions

The effect of occupational exposure to lead on the metabolism of trace metals appears to be limited and concerns mainly their tissue distribution. However, results of the present study indicate that exposure to lead significantly influences blood levels of Cu and Se. Besides, altered levels of CER and HPG were shown. This changes in levels of the acute-phase proteins may be associated with lead-induced modifications of the immune system.

The accumulated data indicate that Fe, Zn, Se and Cu may reduce lead toxicity; thus, an adequate dietary intake of the above-mentioned trace metals is necessary. However, there is no evidence that additional supplementation would be beneficial.
